# A Case of Excision of the Uterus

**Published:** 1811-03

**Authors:** Richard Baxter

**Affiliations:** Surgeon to prisoners of war, on parole at Montgomery, and to the Montgomery and Pool House of Industry


*To the Editors of the Medical and Physical Journal.*

Gentlemen,

Mr. Baxter, a surgeon of great respectability at Montgomery, has very obligingly communicated to me an extraordinary case in midwifery, which I think ought to be preserved; he has favored me with several documents to prove the facts here recorded, but as these do not seem necessary to be published, I content myself with sending you a simple relation of the case, which I doubt not you will readily insert in the next number of the Medical and Physical Journal.

I remain, &c.
SAMUEL MERRIMAN.

*Curzon Street, Feb.* 6, 1811.

---

*A Case of Excision of the Uterus; by* MR. RICHARD BAXTER, Surgeon to prisoners of war, on parole at Montgomery, and to the Montgomery and Pool House of Industry.

MARY Griffiths, a married woman, thirty-seven years of age, residing in the parish of *Guilsfield*, near *Welsh-Pool*, in Montgomeryshire, was delivered of her first child about six years ago; her labour was natural and rather quick; she recovered speedily and perfectly from this confinement, and afterwards enjoyed an uninterrupted good state of health, till the period of her second labour, which began on Tuesday, the first of May, 1810. During that day she had frequent and often strong pains, which continued more or less severe till Wednesday evening about five o'clock, when the child was born, no more difficulty having occurred than commonly happens in a long and what may be called a *laborious* labour.

Though there was no flooding nor other ill symptom, yet as at the end of half an hour the placenta had not separated, the midwife proceeded to extract it, in doing which she employed so much force, that she dragged out the placenta and uterus together, when the placenta immediately separated from its attachment, and the uterus remained without the os externum. The poor woman says, that she repeatedly requested the midwife to desist, as she fancied that she was drawing her inside out, but the midwife persevered in her forcible attempts, till, at length, the patient being sensible of something very large passing from her, believed herself to be delivered of another child. From this period till a considerable time afterwards she knew not what passed, as she fainted, and on her recovery found that there had been a profuse flooding, and that there was a large tumour externally.

No attempt was made to return this tumour, and in this situation she remained three weeks, when, with the greatest difficulty, she walked to *Welsh-Pool*, a distance of nearly three miles, where she received medical attendance. What was done for her I have not been able to learn, but the exertion of walking brought on a very violent inflammation of the parts; during the first fortnight of which there had been a great a great sloughing of the internal (now external) coats of the uterus, attended with a very fœtid ichorous discharge.

A fortnight after this, and five weeks from the accident, she was sent a most miserable object to the *Montgomery and Pool House of Industry*, there to linger out, as it was supposed, the short remaining period of her life, rather than from a hope that any relief could be afforded her.

In this deplorable and debilitated state she came under my care. I found on examination that reduction of the tumour was now impracticable, and that extirpation was the only chance left to put an end to her sufferings. This operation I therefore determined on, and performed as follows; having first submitted the patient to the inspection of Dr. E. Jones, an eminent physician in this neighbourhood, "who had not the smallest doubt that the case was a complete inversion of the uterus."

I passed a flat seton needle through the vagina, edgeways, in the direction of the longitudinal fibres; then dividing the ligature, I tied each half moderately tight, and fearful lest these might slip, I tied another ligature* round the whole, close above the former. I then divided the vagina with a scalpel immediately below the lower ligatures. The hæmorrhage that ensued was trivial, and that, principally, from the introduction of the needle. By this operation the whole of the uterus and at least an inch and a half of the vagina were extirpated. In about nine days, the lower ligatures came away, but the upper one remained much longer. The patient in a fortnight began to increase in her flesh, and in six weeks from the operation was perfectly recovered; so much was she improved in her appearance, that those who saw her but a few weeks before scarcely knew her again.

On making an incision through the tumour thus extirpated, commencing at the vagina, and continuing it along the whole length and through the body of the uterus, no vestige of a cavity remained, not even to admit of a fine probe; for from the great inflammation that ensued, before *any proper medical advice was afforded*, the parts were become so completely diseased, as to preclude any possibility of disco-

* I much repented having used the upper ligature, as it very much retarded the cure, and I found the two inferior ones amply sufficient. The great thickness and scirrhosity of the vagina, prevented the ligature which was passed round the whole from penetrating through its substance, and occasioned great delay, till I afterwards drew the strings much tighter, when it came away and left the parts in their present state.

vering the ovaria or fallopian tubes : it was indeed one solid schirrous mass, the effect of the inflammation.

Soon after this patient was discharged from the *Montgomery and Pool House of Industry*, she went to a distant part of the country, and I did not see her for some months; I have, however, lately examined her, and made the following remarks.

Her present state of health is nearly as good as prior to her delivery, except that she complains of her breath being shorter than formerly.

Her stools and urine are natural, and were so most of the time. Two days after the operation I thought it proper to give her some castor oil, which greatly relieved her; she took very little other medicine, excepting the cinchona, during the cure, neither was the symptomatic fever at all considerable.

She perceives, at times, an unusual emptiness in the lower region of the belly, but no appearance or feel of the descent of the bowels. For a few days after the operation, she felt as if the wind was passing into her abdomen, particularly every time the parts were exposed in order to have fresh applications made to the wounds.

She had an appearance of menstrual discharge on the 24th of October last, nearly five months from her delivery, and more than two months after she was discharged as cured, and the same circumstance has happened once since. She did not feel the usual fulness of her breasts nor any other symptoms to indicate the approach of this appearance. The discharge was not so copious nor so florid as usual. These are the only times she has observed any thing like a menstrual discharge; but for a short period subsequent to the operation, she had a mucous discharge, which had altogether ceased.

Upon examining the parts, when the finger is passed about half its length, there may be perceived an hiatus at the termination of the vagina, of an irregular figure, but not large enough to admit a crow quill.

Cases of excision of the uterus are very rare; one is related in Duncan's "Annals of Medicine" for 1799, but this gives not the least clue as to the mode of operation. Another is recorded by Professor Wrisberg, a translation of which has been published by Dr. Hooper, in the "London Medical Review and Magazine," vol. 5, for the year 1801; in this the inverted uterus was cut away by the midwife.

Heister thinks, that even if the inverted uterus were to be secured by a ligature, and extirpated, it would not save the life of the woman, for, he says, Ruysch gives us an example of this disorder, in which the surgeon attempted to relieve lieve the patient, by making a ligature and cutting off the prolapsed body of the womb, but his design miscarried and the woman died soon after.

Palfyn* does not believe that the uterus itself has ever been amputated; he says, "Au reste ce qui est rapporté par quelques auteurs de l'amputation de la matrice en entier, doit être reputé pour un conte." He conceives that the tumour supposed to be the prolapsed uterus, is produced by an elongation of the internal membrane of the vagina. His words are, "La membrane interne du vagin est quelquefois tellement relachée par les humeurs superflues qui l'abreuvent, qu'elle descend beaucoup plus bas que la partie honteuse, et qu'elle se montre au-dehors; ce qui les anciens ont pris pour une descente de matrice."

In Palfyn's second volume, is a representation of the uterus divided lengthways, pl. xv. fig. 3, which gives an exact appearance of the tumour I removed, with this difference, that in Palfyn's figure, the cavity of the uterus is shewn, but in mine it was obliterated; what he describes as "*les fibres charnues de la matrice*" are an exact resemblance.

Inversions of the uterus are, I fear, more common than is usually known, or at least acknowledged; four cases have occurred within my own knowledge, two of which being speedily reduced were attended with no ill consequences; the third was the case which I have just related; and the fourth has since happened at Bishop's Castle, where the midwife tore off the uterus, mistaking it for a mole, and the poor woman instantly died. RICHARD BAXTER.

---

*To the Editors of the Medical and Physical Journal.*

Gentlemen,

If you think the inclosed Tables, collected with some labour for my own use, likely to afford either amusement or information to your readers, they are very much at your service. In return I should be happy to receive from any of your intelligent correspondents, replies to the annexed queries. I remain, &c.

*Feb.* 11, 1811. OBSTETRICUS.

---

* In his "Anatomy," edited by Petit. Palfyn has two works on the subject of anatomy. *Description anatomique des parties de la femme qui servent à la génération, avec le Traite des monstres de* Fortunio Liceti, &c. 4to. 1708, and in Flemish, 1724. Leyden.—*Anatomie Chirurgicale, ou description exacte des parties du corps humain, avec des remarques utiles aux Chirurgiens dans la pratique de leur Art*, 8vo. 1710, 1718, Leyd.—8vo. in German, 1717. Leipsic. Ed.

